# The Multi-Therapeutic Role of MSCs in Diabetic Nephropathy

**DOI:** 10.3389/fendo.2021.671566

**Published:** 2021-06-07

**Authors:** Yi Wang, Su-Kang Shan, Bei Guo, Fuxingzi Li, Ming-Hui Zheng, Li-Min Lei, Qiu-Shuang Xu, Muhammad Hasnain Ehsan Ullah, Feng Xu, Xiao Lin, Ling-Qing Yuan

**Affiliations:** ^1^ Department of Metabolism and Endocrinology, National Clinical Research Center for Metabolic Diseases, Hunan Provincial Key Laboratory of Metabolic Bone Diseases, the Second Xiangya Hospital, Central South University, Changsha, China; ^2^ Department of Radiology, the Second Xiangya Hospital, Central South University, Changsha, China

**Keywords:** exosomes, therapy, diabetic nephropathy, hyperglycemia, mesenchymal stem cells

## Abstract

Diabetic nephropathy (DN) is one of the most common diabetes mellitus (DM) microvascular complications, which always ends with end-stage renal disease (ESRD). Up to now, as the treatment of DN in clinic is still complicated, ESRD has become the main cause of death in diabetic patients. Mesenchymal stem cells (MSCs), with multi-differentiation potential and paracrine function, have attracted considerable attention in cell therapy recently. Increasing studies concerning the mechanisms and therapeutic effect of MSCs in DN emerged. This review summarizes several mechanisms of MSCs, especially MSCs derived exosomes in DN therapy, including hyperglycemia regulation, anti-inflammatory, anti-fibrosis, pro-angiogenesis, and renal function protection. We also emphasize the limitation of MSCs application in the clinic and the enhanced therapeutic role of pre-treated MSCs in the DN therapy. This review provides balanced and impartial views for MSC therapy as a promising strategy in diabetic kidney disease amelioration.

## Introduction

Diabetic nephropathy (DN) is one of the most common complications of Diabetic Mellitus (DM) (1). Parallel with the rising global prevalence of diabetes, DN often occurs after diabetic retinopathy, another microvascular complication of DM, presenting symptoms after 10 to 15 years of diabetes (2–4). The characteristics of DN are concluded as persistent proteinuria, reduced total glomerular filtration rate, raised arterial blood pressure, fluid retention, and shrunken kidney size (5–7). With intractable and refractory pathological progression, DN tends to progress into chronic kidney diseases (CKD). Almost half of people with type 2 diabetes will suffer CKD, as do approximately one-third of type 1 diabetes patients (8). Additionally, CKD always ends with end-stage renal disease (ESDR), leading to an extremely high rate of kidney transplantation and death (9–11). Up to now, the current medical treatment for DN still relies on pharmacological treatment aimed at glycaemic and blood pressure control, as well as kidney protection. Typical drugs like Chinese herbal medicine (12) and renin-angiotensin system-blocking medication (13) play a role while rarely change the outcome of DN. A study shows that 60.3% of patients being diagnosed with stage 4 CKD with DN rapidly progressed to ESRD or death (10.9%) after the treatment of angiotensin II type 1 receptor blocker (ARB) drugs and Rheum (13). Another data show over 200,000 deaths ascribed to advanced CKD/ESRD from 2003 to 2017 in the United States, and even with effective drug treatment, 25% of people with type 2 diabetes and DN eventually develop ESRD (11). Despite this, poor prognosis of ESRD can be alleviated with early diagnosis and treatment of chronic kidney diseases (9). Thus, the poor prognosis of DN drives the efforts of many scientists to discover pathological mechanisms and effective therapy of DN.

Recently, increasing attention is being focused on mesenchymal stem cell (MSC) therapy. MSCs are specific types of cells under exploration for treatment of human diseases and have been found in tissues including adipose tissue (14), peripheral blood (15, 16), dental pulp (17), bone marrow (18), and neonatal tissues, especially in parts of the placenta (19) and umbilical cord (20, 21). The definition of MSCs involves three features: Self-renewal ability; Multi-differentiation potential; Specific surface biomarkers (22, 23). It had been shown that MSCs present with the capacity for self-renewal (24). Additionally, MSCs can differentiate into multiple cell types like chondroblasts (25), osteoblasts (26) adipocytes (27), and neuron-like cells (24) under specific induction. Over 95% of MSCs express surface markers CD73, CD90, CD105, while MSCs are negative for the expression of CD14, CD34, CD45, and human leukocyte antigen-DR (HLA-DR) (22, 28). Additionally, MSCs are capable of excreting small molecules, such as cytokines and exosomes. Owing to these unique features, MSCs appeal to researchers. Up to now, increasing numbers of studies concerning the therapeutic role of MSCs are ongoing. It had been reported that MSCs can alleviate disease progressions like stroking (29), myocardial infarction (30), and tumor (31). Furthermore, some clinic tests had made progress in the potential therapeutic role of MSCs.

MSCs derived exosomes, lipid membrane micro-vesicles with the size of 30-150nm, have been found to play a significant role in MSC therapy. Genetic molecules, including RNA (32, 33), and proteins (34, 35) can modulate micro-environments and epigenetic phenomena of organisms both in normal or pathological conditions. Thus, exosomes carrying numbers of these substances (36, 37), shuttling between cells and tissues, can transfer signals or materials and mediate micro-environmental communication in several types of diseases (38–40). Other studies have reported that cargo within MSCs derived exosomes mediates therapeutic approaches of diverse types of diseases, such as tumor (41), infections (42), metabolic diseases (43), and immune diseases (44).

Research related to MSC therapy is ongoing. Exploration concerning the therapeutic role of MSCs in DN, especially MSCs derived exosomes, are limited. This review covers the latest progress of MSCs treatment of DN, emphasizing the role of MSCs derived exosomes in these mechanisms and potential options for future therapies.

## The Therapeutic Mechanisms of MSCs in DN

**Figure 1:**
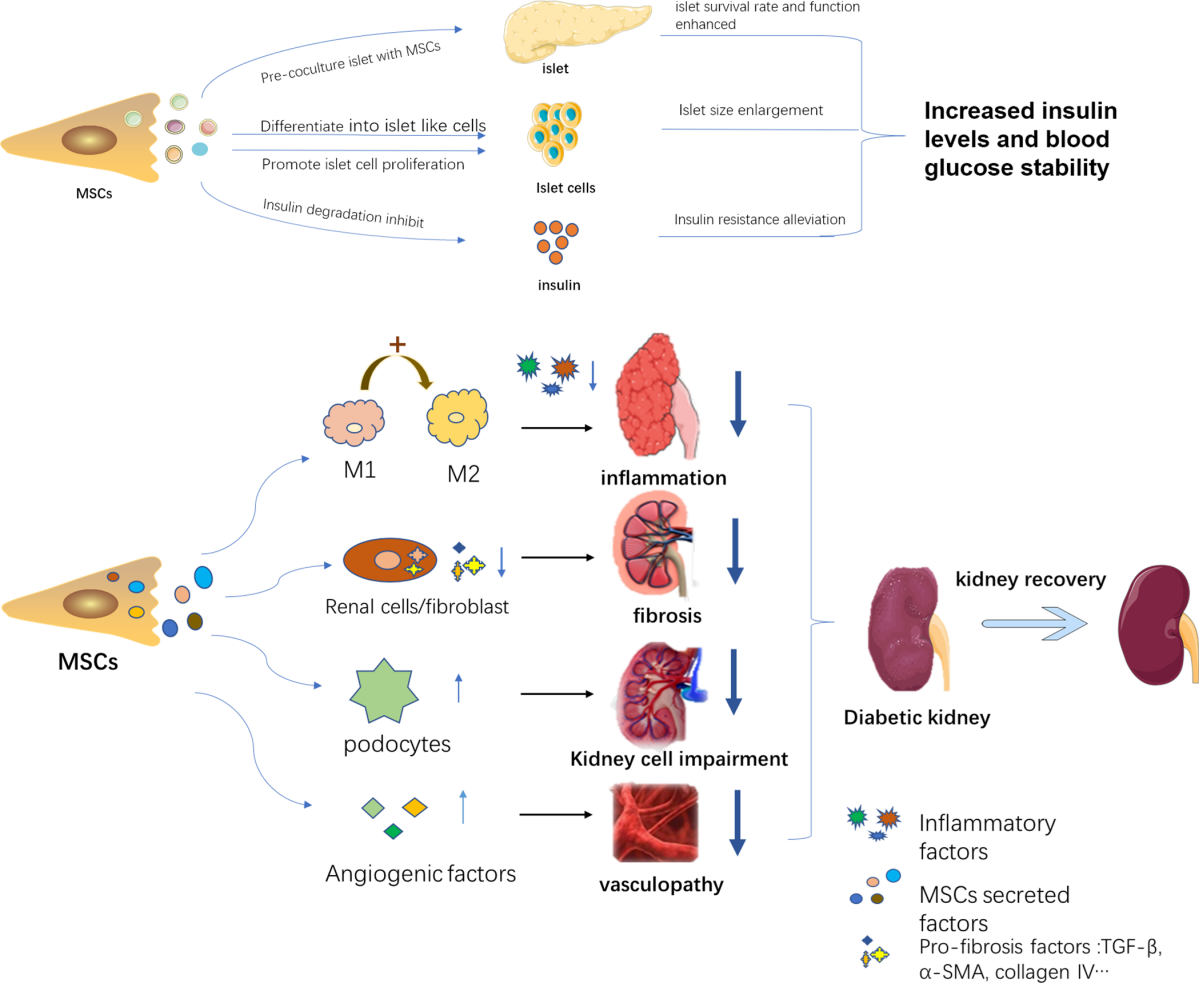
The therapeutic mechanisms of MSCs in DN. MSCs alleviate DN progress in two pathways: 1. Decreasing blood glucose through islet function recovery, islet cell proliferation, and insulin sensitivity improvement. 2. Acting against kidney inflammation, fibrosis, and protect kidney-related cells and promote angiogenesis.

DN often occurs during persistent high blood glucose in a DM patient, proceeding into CKD and ESRD. Hyperglycemia and kidney dysfunction are both therapeutic targets to alleviate the progress of DN. Accordingly, MSCs play a part in DN treatment mainly in two pathways, including hyperglycemia control and kidney impairment alleviation. MSCs can alleviate high blood glucose by promoting regeneration of islet cells and reducing insulin resistance, as well as improving islet function, thereby lessening the kidney injury resulting from high blood glucose. MSCs can also directly rescue kidney damage *via* diverse mechanisms. A bunch of studies demonstrated a phenomenon that MSCs treatment improved renal function by acting against inflammation, fibrosis, apoptosis as well as promoting angiogenesis. Only a portion of these mechanisms has been revealed, while the majority remains to be explored. The detailed aspects are shown in **Figure 1**.

### The Role of MSCs in Blood Glucose Control

#### MSCs in the Regeneration of β Cells

MSCs present with potent potential for the regeneration of β cells. MSCs can differentiate into insulin-producing cells. Pan et al. found that the notch signal pathway of MSCs was highly inhibited under high glucose treatment *via* the methylation of notch-related genes, which suggested the directional differentiation of MSCs into functional β-cells (45). Another study also implied that insulin levels in circulation together with insulin-producing cells were increased after MSCs transplantation in diabetic mice model, suggesting MSCs are capable of differentiating into β-like cells (46). The types of MSCs that differentiate into insulin-producing cells are not limited, while the capability is not the same. Research indicated that although both BM-MSCs and subcutaneous adipose-derived MSCs can differentiate into islet-like clusters, BM-MSCs are superior to MSCs derived from adipose tissues in this process (47). Wharton’s jelly-derived MSCs (WJ-MSCs), a type of perinatal stem cells with specific cell surface biomarker of EphA2, had also shown great potential in regeneration medicine (48). Previous research has focused on the transplantation of WJ-MSCs that had differentiated into islet-like cells *in vitro* (49, 50). However, a recent study revealed that even undifferentiated WJ-MSCs can migrate to the pancreas and differentiate into insulin-producing cells (51). Another research also reported a protocol to differentiate WJ-MSCs into pancreatic insulin-producing cells (52). At the same time, a clinical trial demonstrated that WJ-MSCs progressively decreased the glycated hemoglobin levels, fasting glucose level, and fasting serum C-peptide levels (53). A meta-analysis concerning six studies of WJ-MSCs therapy in 172 diabetic patients had demonstrated that WJ-MSC transplantation could improve HbA1c%, as well as C-peptide levels in both T1DM and T2DM (54). However, the number of included studies and the patients involved in most cases were quite limited, so further clinical studies are required to investigate the therapeutic efficacy of WJ-MSCs. Furthermore, MSCs promote endogenous β cell proliferation and replication. Apelin overexpression in MSCs leads to a significant expansion of β cell numbers and total pancreatic ß cell mass as well as enlarged islet size, implying the pro-proliferation effect of MSCs (55). Additionally, PI3K/Akt pathway inhibitors blocked the proliferation of β cells mediated by MSCs-conditioned medium, suggesting MSCs secretion induced β cell replication *via* the PI3K/Akt signal pathway (56). By these two ways, MSCs effectively promote islet β cell regeneration, thereby decreasing high blood glucose and its related hyperglycemia index.

#### MSCs in the Insulin Resistance

MSCs are also involved in the improvement of insulin sensitivity. Insulin resistance is another crucial point in the DM pathological process, especially in type 2 diabetes. Insulin resistance results in decreased insulin sensitivity, causing blood glucose to hardly back to a normal level and persistent hyperglycemia. Si et al. revealed that infusion of MSCs ameliorated hyperglycemia and proposed for the first time MSC therapy for improvement of insulin sensitivity (57). While the glucose-decreasing effect caused by a single infusion of MSCs was maintained only for a few days, further exploration found that multiple intravenous MSCs infusions reversed hyperglycemia and kept glycemia within normal levels (58, 59). As mentioned above, apelin may play vital roles in hyperglycemia remittance. Not only does it promote β cell proliferation, but apelin also increases insulin sensitivity. One study found that apelin-transduced WJ-MSCs rats shared faster glucose disposal and improved glucose tolerance compared to a placebo group (55). Several mechanisms had been reported to explain such improvement. Muscle mitsugumin 53 (MG53), a newly identified muscle-specific protein, is one pivotal element of insulin resistance in type 2 diabetes by participating in the insulin degradation process through insulin receptor substrate-1 (IRS-1) and the p-AKT pathway. MSCs infusion significantly inhibited MG53 elevation, subsequently restraining insulin-related factor degradation and alleviating insulin resistance (60). A clinical comparative study showed that DM patients with MSCs transplantation had an improved insulin sensitivity index, consequently resulting in a recession in demand of insulin doses. The declined area under curve (AUC) of 2^nd^phase C-peptide response and restoration of IRS-1 expression in patients treated with MSCs provided further evidence for this therapy (61). Inflammatory cytokines and immune regulation also contribute to insulin resistance. Elevated inflammatory factors took part in insulin receptor destruction, exacerbating insulin resistance to a large extent. Sun X and colleagues observed raised NLRP3, L-1β, IL-18, and TNF-α expression in a type 2 diabetes mouse model and that this elevation could be blocked by MSCs injection. The result implied MSCs could reduce inflammatory activities by downregulating the NLRP3-mediated inflammation pathway, accordingly alleviating insulin resistance (62). Macrophage polarization is another anti-inflammation approach for diabetes that enhances insulin sensitivity. Two distinct populations of macrophages have been discovered, including pro-inflammatory macrophages (M1) and anti-inflammatory macrophages (M2). The research revealed that macrophages could be transformed from M1 to M2 in adipose tissue by the MSCs-activated IL-4R/STAT6/STAT3/PPARγ axis as well as MSCs-secreted monocyte chemoattractant protein-1(MCP-1) and IL-6, improving inflammation and insulin sensitivity (63, 64). These results imply anti-inflammation is a crucial point in improving insulin sensitivity.

Immune regulation of MSCs also participates in blood glucose control *via* other mechanisms. MSCs provide a suitable environment for β cell survival through the regulation of some immune factors and cells. Boumaza et al. demonstrated that T cell cytokines were altered and the frequencies of CD4^+^/Foxp3^+^ and CD8^+^/Foxp3^+^T cells increased under MSCs treatment, enhancing β cell function (65).

As mentioned above, MSCs treatment could attenuate insulin resistance by decreasing inflammatory factors, regulating macrophage polarization and immune function.

#### MSCs in the Islet Dysfunction

There is additional evidence to show MSCs are competent to decrease blood glucose. In DM treatment, especially for type 1 diabetes, islet dysfunction is the key therapeutic focus. Traditional treatment around islet transplantation had been investigated for decades, but outcomes are unpredictable and ambiguous. Remarkably, the latest research revealed that MSCs can improve the function and survival rate of transplantation islets. Montanari et al. found that insulin secretion of the free islet was enhanced under MSCs treatment *via* the adhesion molecule N-cadherin, which improved survival and function of islets of Langerhans (66). WJ-MSCs, which contribute to the regeneration of β cells, were able to repair the destroyed islets as well by reducing the severity of insulitis in DM mice (51). Pre-culturing islets with a mixture of MSCs products put forward a perspective of cell-free therapy to improve clinical islet transplantation outcomes (67, 68). At the same time, researchers had demonstrated annexin A1 as playing an important role in this pathway (69). Another study also discovered enhanced glucose homeostasis under the co-transplantation of MSCs together with islets (70). Furthermore, such treatment effect of MSCs on islet can be improved under pre-hypoxic conditions (71). These results reveal that MSCs are beneficial for islet function improvement, suggesting MSC therapy as a prospect for hyperglycemia recovery.

### The Role of MSCs in Kidney Impairment

The hyperglycemia control and islet cell protection in DN treatment work as effective ways to delay diabetes caused kidney impairment. However, direct protection and repairment toward kidney function are of more significance and efficiency. It has been shown that MSCs can regulate the immune environment, reducing fibrosis formation, and promoting angiogenesis. Additionally, the majority of these processes are accomplished by exosome-mediated paracrine function, which suggests that exosomes play a pivotal role in kidney function recovery (72).

#### The Role of MSCs in Anti-Inflammation and Anti-Fibrosis

The pathogenesis of DN is currently understood to be multifactorial, where inflammation appears to be relevant in the DN process, leading to metabolic disorder. Increasing research concerning inflammatory cell infiltration as well as pro-inflammatory cytokines secretion in DN pathogenesis gives a clue for DN treatment.

MSCs directly regulate immune cell migration and filtration, thereby reducing inflammatory activation. It is well-known that macrophages play an important role in the inflammatory process, and considerable research has focused on the macrophage. It had been demonstrated that MSCs-derived HGF inhibited MCP-1 expression to prevent macrophage infiltration (73). Lee et al. also found MSCs were associated with macrophage recruitment *via* expressing markers like C-C motif chemokine ligand 2 (Ccl2), vascular cell adhesion molecule-1 (VCAM1), and intercellular adhesion molecule-1 (ICAM1) (74). Similarly, research showed that the intravenous injection of MSCs reduced renal CD68^+^ macrophage infiltration and inflammatory cytokine expression in the kidney of diabetic rats, and the fibrosis had been ameliorated (75). Meanwhile, the inductive effects of MSCs in macrophage polarization play a part in the impaired kidney as well. Lee’s team realized increased expression of Arg1 in human umbilical cord blood MSCs could inhibit M1 polarization of macrophage, which decreased inflammatory factor secretion. Conditioned medium with human umbilical cord blood MSCs were able to rescue DN-induced mitochondrial mass reduction and mitochondrial reactive oxygen species (ROS) production compared to original adipose MSCs, which suggests that these effects were limited to umbilical cord blood-derived MSCs (74). Transcription factor EB (TFEB) expression was also found to be related to macrophage polarization. A study revealed that MSCs elicited macrophage transformation into the M2 phenotype *via* a TFEB-dependent mechanism. The transcription of TFEB activated the restoration of lysosomal and autophagy as well as mitochondrial bioenergetics of macrophages, which inhibited the pro-inflammation reaction (76). All these results suggested MSCs are capable of impacting macrophage function to inhibit inflammation activity.

The fibrosis and epithelial-mesenchymal transformation (EMT) had been regarded as a typical pathological change in DN as well, resulting in serious glomerular sclerosis and impaired filtration function. Research concerning the therapeutic role of MSCs in anti-fibrosis is ongoing and has achieved some promising results.

Interestingly, it seems like fibrosis and inflammation share several common pathways, as the treatment with MSCs tends to ameliorate fibrosis and inflammation together. Except for decreased inflammatory factors, collagen IV, α-SMA, and TGF-β in the kidneys of DN rats were decreased after MSCs treatment in the study of Xiang et al, which suggested MSCs can inhibit fibrosis as well (77). Another study had demonstrated that Lipoxin A4 played a key role both in inflammation and fibrosis progression in DN pathogenesis. MSCs-derived Lipoxin A4 could reduce TGF-β as well as Smad2/Smad3 expression, a group of key factors attributed to extracellular matrix dysfunction, to rescue the fibrosis process. Meanwhile, three pro-inflammatory cytokines were decreased after MSCs-Lipoxin A4 injection, suggesting the pro-inflammatory actions had been inhibited by MSCs-derived Lipoxin A4 (78).

In conclusion, MSCs inhibit inflammatory reactions *via* impacting immune cell filtration. Additionally, EMT and fibrosis processes are delayed together with anti-inflammation of MSCs in DN.

#### The Role of MSCs in Podocytes Protection

Podocytes are regarded as the third layer of kidney filtration membrane structure, preventing protein loss from urine. Research has demonstrated that podocytes were decreased under persistent high glucose stimulus, which leads to albuminuria and proceeded to injure kidney function (79, 80). Thus, podocyte injury is an obvious pathological phenomenon in diabetes kidneys.

Several studies discovered that MSCs injection and transplantation could attenuate albuminuria and improve kidney function, which suggests that MSCs protect podocytes from dysfunction and injury. An animal study had demonstrated that rats treated with MSCs showed a suppressed increase in creatinine clearance rate and urinary albumin-to-creatinine ratio. Furthermore, the MSCs treatment reduced the loss of podocytes and podocyte markers and increased podocyte survival factor BMP-7 secretion (81). Since MSCs had been demonstrated to treat diabetic nephropathy, something must exist to help MSCs in this process, no matter from other mechanisms or MSCs themselves. Sun et al. revealed that stem cells from bone marrow relieved high glucose-induced podocyte apoptosis in combination with miR-124a *via* inhibiting the notch signal pathway (82). In a further study, they found that overexpressing miR-124a decreased the ROS production as well as cleaved caspase-3, bax, bcl-2, LC3-II/I, and p62 levels. These results suggested the activity of oxidative stress and autophagy of podocytes were significantly reduced by MSCs interfering together with miR-124a. Moreover, other researchers found secreted materials from MSCs also function in the treatment process. Li D and the team screened candidate factors in MSCs-conditioned medium and found that EGF levels were significantly increased, corresponding with lower podocyte apoptosis. At the same time, blocking of EGF decreased the therapeutic effects of MSCs-conditioned medium (83). This suggested that EGF together with MSCs could be regarded as a therapeutic target of DN progression.

To conclude, MSCs treatment can attenuate podocyte oxidative stress as well as podocyte death, thereby rescuing kidney dysfunction and slowing down the process of DN.

#### The Role of MSCs in Pro-Angiogenesis

Tissue reparation and neo-angiogenesis is another essential process in kidney renovation. Researchers found that medium conditioned with MSCs-secreted factors could induce angiogenesis.

Human embryonic MSCs have been found to rescue vascular damage in rats with CKD, and researchers thought that the conditioned medium of MSCs might make efforts in protecting vascular damage. The proteome profile of embryonic MSCs-conditioned medium showed that the presence of several gene products plays a role in angiogenesis and this effect had been subsequently identified in CKD rats. It had been shown that the average tube length was significantly increased in an angiogenesis assay after treatment with MSCs-conditioned medium, suggesting the MSCs-conditioned medium can promote vascular regeneration in the kidney (84, 85). However, this research failed to prove this effect was mediated by exosomes.

### The Role of MSCs Derived Exosomes in DN

Exosomes, vesicles secreted by almost all types of cells, had been revealed to play a significant role in MSC therapy in DN. MSCs derived exosomes are involved in the alleviation of DN progress through aspects previously mentioned, including hyperglycemia control and kidney function protection.

#### The Role of MSCs Derived Exosomes in Blood Glucose Control

MSCs derived exosomes were found to alleviate insulin resistance and directly regulate glucose metabolism by induction of autophagy (86). Qin He and colleagues revealed MSCs derived exosomes participated in glucose homeostasis *via* autophagy-related AMPK pathway inhibition. In their research, the expression of glycolytic enzymes and lipolytic enzymes were increased after MSC-exosome treatment, whereas hepatic gluconeogenic enzymes were decreased; This suggests that MSCs derived exosomes were involved in the glucose metabolism to down-regulate hyperglycemia (87). Furthermore, MSCs derived exosomes increased the regulatory T-cell population and their products without a change in the proliferation index of lymphocytes in patients with moderate autoimmune type 1 diabetes, providing a suitable environment for β cell survival (88). Thus, MSCs derived exosomes ameliorate hyperglycemia *via* improved insulin sensitivity and β-cell function.

#### The Role of MSCs Derived Exosomes in Kidney Impairment

Exosomes derived from MSCs present a crucial role in kidney function repairment. Xiang et al. revealed that human umbilical cord-derived MSCs reduced inflammation both in DN rats and kidney cells. The mRNA expression of IL-6, IL-1β, and TNF-α was elevated in DN rats but was significantly decreased in MSCs-treated groups. To further identify these effects, Xiang et al. co-cultured MSCs derived exosomes with high-glucose-treated kidney cells, which included HK2 cells, NRK-52E cells, and hRGE cells; Results showed that MSCs derived exosomes suppressed high glucose-induced production of TGF-β, IL-6, IL-1β, and TNF-α in a dose-dependent manner. Moreover, several factors such as epidermal growth factor (EGF), fibroblast growth factor (FGF), hepatocyte growth factor (HGF), and vascular endothelial growth factor (VEGF) were detected in MSCs derived exosomes, which suggests the anti-inflammatory effect was mediated by MSCs derived exosomes (77). Some studies found exocellular vesicles especially exosomes derived from MSCs had played a significant role in anti-fibrosis mechanisms. Some cohorts found DN mice treated with MSCs-derived extracellular vesicles presented improved kidney fibrosis, which suggested some specific patterns of miRNAs were involved in fibrosis (89). To be more specific, Ling Zhong’s team revealed that MSCs-derived micro-vesicles shuttled miRNA-451a to down-regulate P15 and P19 expression, which assisted in restarting the cell cycle and slowed down the process of EMT, thereby regulating kidney fibrosis in DN (90). Other anti-fibrosis mechanisms had been revealed concerning the matrix-related proteins. MSCs treatment significantly decreased the proliferation of mesangial cells and upregulated matrix metalloproteinase (MMP) levels, which was related to extracellular matrix protein accumulation. MSC injection blocked myofibroblast trans-differentiation that resulted in reduced TGF-β1, fibronectin, and collagen I; These regulatory effects could be abolished by exosome consumption (91). This suggested that exosomes played a key role in ameliorating DN renal fibrosis. Additionally, autophagy had been shown to participate in the process of fibrosis development. One study found that MSCs derived exosomes reversed the diabetes-stimulated autophagy-related reduction in gene expression. Exosomes from MSCs could ameliorate the overexpression of TGF-β and fibronectin that were induced by autophagy inhibition, thus attenuating fibrosis, suggesting that these exosomes are capable of activating autophagy to protect renal function (92).

MSCs derived exosomes are involved in podocyte protection as well. Exosomes originating from adipose stem cells containing microRNAs powerfully impeded high glucose-induced migration and injury of podocytes. Remarkedly, several MSCs-derived exosomal microRNAs were found to participate in kidney cell protection. Adipose-derived stem cells secreted exosomes to adjust the survival of podocytes in the DN process. Mao et al. had discovered that microRNA-let-7a plays a protective role in renal cell apoptosis by targeting ubiquitin-specific protease 22 (USP22). Both elevated exosomal miR-let-7a or silenced USP22 reduced the apoptosis of renal cells and improved kidney function (93). Additionally, it had been demonstrated that the miR-251-5p inhibitor counteracted the improvement conferred by MSC exosomes on high glucose-induced proliferation inhibition and migration promotion of podocytes; And the miR-251-5p mimics significantly reversed the EMT process of the podocyte, suggesting exosomal miR-251-5p plays a role in podocyte protection (94). Meanwhile, miR-26a-5p took part in this process by targeting TLR4. Overexpression of miR-26a-5p inactivated the NF-κB pathway and downregulated vascular endothelial growth factor A (VEGFA) (95). Exosomal miR-16-5p from human urine-derived stem cells had been reported to alleviate DN *via* increasing podocyte viability and decreasing the rate of apoptosis. Overexpressed miR-16-5p in human urine stem cells significantly improved proteinuria as well as kidney function index (96). All this research concluded that miRNA could be adjusted to control the DN condition. MSCs derived exosomes are of great importance in the podocyte’s protection, providing a novel perspective for DN therapy.

Another researcher investigated the pro-angiogenesis function of exosomes from MSCs. They repeatedly demonstrated the pro-angiogenesis function of MSCs-conditioned medium and identified that this potential was mediated by exosomes (97). Similarly, urine stem cell-derived exosomes contained increased VEGF, TGF-β, and angiogenin, which were reported to be involved in angiogenesis and cell survival (98). Up to now, most of these studies were limited to factor level detection, making the specific pro-angiogenesis mechanisms unknown. Notably, even though VEGF factor function had been verified to promote angiogenesis in other disease models, the function of VEGF is still undefined in DN since it had been reported to increase glomerulus permeability and proteinuria (99–101). There are few studies focused on the mechanism of VEGF derived from MSCs derived exosomes in DN models, which make the function of VEGF still puzzling in the DN process.

Overall, the specific functions of MSCs from different origins in kidney protection are covered in **Table 1**. The factors released by MSCs as well as involved DN models in kidney function recovery are listed.

**Table 1:** The detailed function of MSCs secreted factors in diabetic nephropathy.

MSCs Original	Model	Secreted Factors	Function	Reference
Human umbilical cord	DN rats; HK2 cells, NRK-52E cells, hRGE cells	EGF, FGF, HGF, VEGF	Anti-inflammation and fibrosis	(77)
Human umbilical cord	Rhesus macaque;HK2 cells	IL-16	Anti-inflammation and fibrosis	(102)
Bone Marrow	DN rats;	HGF	The expression of MCP-1 could be inhibited *via* MSCs secreted HGF, thereby reducing macrophages infiltration, and pro-inflammatory cytokines	(73)
Human umbilical cord	Mice; RAW264.7 cells	Arg1	Arg1 suppress M1 polarization and improve macrophage mitochondrial function, thereby inhibiting inflammation	(74)
Bone Marrow	DN rats; Peritoneal macrophages	–	suppressed renal macrophage infiltration and inflammatory cytokine secretion	(75)
Bone Marrow	DN mice; Peritoneal Mφ	TFEB	TFEB mediate macrophage transfer into M2 to promote anti-inflammatory reaction	(76)
Bone Marrow	DN rats; Glomerular mesangial cell	Lipoxin A4	Lipoxin A4 suppress fibrosis viatargeting TGF-β/smad axis; Anti-inflammation	(78)
Human umbilical cord	DN mice; HK2 cells	miR-451a	Down the expression of α-SMA, P15INK4b, and P19INK4d to inhibit EMT process and restart cell cycle, thereby slowing fibrosis.	(90)
Mouse umbilical Cord	DN mice; Mouse mesangial cell	—	Exosomes from MSCs reduced the fibronectin and collagen expression *via* inhibiting myofibroblast trans-differentiation triggered by TGF-β1 and cell proliferation mediated by PI3K/Akt and MAPK signaling pathways and elevating the levels of MMP2 and MMP9.	(91)
Bone Marrow	DN rats	—	MSCs-exosomes increased autophagy markers mechanistic target of rapamycin (mTOR), Beclin-1 as well as light chain-3 (LC-3) to activate autophagy, thus improve renal fibrosis.	(92)
Bone Marrow	DN rats; Renal cell	miR-let-7a	Increased miR-let-7a in MSCs-exosomes reduced blood urea nitrogen (BUN) and serum creatinine (SCr), blood lipid-related indicators total cholesterol (TC) and triglyceride (TG), renal cell apoptosis by repressing USP22 expression	(93)
Bone Marrow	DN Rats;	—	MSCs injection promoted podocytes to express higher levels of BMP-7, and improved kidney function	(81)
Bone Marrow	DN rats; Murine podocytes	miRNA-124a	MSCs combined with miRNA-124a down-regulate the expression of Notch1, NICD, Hes1 and Delta to reduce podocytes apoptosis.	(82)
Bone Marrow	podocytes	miRNA-124a	Overexpression of miRNA-124a decreased the intensity of oxidative stress and autophagy of podocytes *via* the PI3K/Akt/mTOR pathway	(103)
Umbilical Cord	DN rats;	—	MSCs up-regulated anti-apoptosis proteins expression and suppressed apoptosis signal regulating kinase 1 and P38 MAPK	(104)
Adipose	DN mice podocyte	EGF	EGF increased in MSCs condition medium to attenuate podocyte apoptosis.	(83)
Adipose	DN mice MPC5 cells	miR-215-5p	Exosomes from adipose stem cells containing mir-215-5p to inhibit EMT of podocytes *via* zinc finger E-box-binding homeobox 2 (ZEB2)	(94)
Adipose	DN mice MP5 cells	miR-26a-5p	Adipose MSCs-exosomes containing mir-26a-5p attenuate kidneys cells injury *via* targeting Toll-like receptor 4 (TLR4).	(95)
urine	DN rats podocytes	miR-16-5p	Overexpression of miR-16-5p in urine stem cells exosomes inhibited VEGFA expression to confer protective effects on human podocytes	(96)
Urine	DN rats podocytes	VEGF, TGF-β1, angiogenin	The VEGF, TGF-β1, and angiogenin might be related to angiogenesis.	(98)

## Limitation and Potential of MSCs Therapy in DN

### Limitation of MSCs Therapy

MSCs present an excellent therapeutic effect on renal function alleviation, which offers the desired perspective for novel DN therapy. However, the progression of passing MSCs therapy from the bench to the bedside has been very slow for several reasons. The quantity and quality of MSCs are the most challenging for clinic application. For the quantity, even though the procedure for MSCs isolation and expansion into a nonclonal population of stromal cells had been standardized according to the International Society for Cell & Gene Therapy (ISCT), MSCs originate from different donors or even different tissues have diverse proliferation rates and capability. Meanwhile, every nonclonal population of MSCs may contain a different proportion of stem cells, which may affect the biological properties of the total population. Therefore, the percentage of stem and progenitor cells in each batch of MSCs must be evaluated exactly before being used in patients (105). For the quality, MSCs ex vivo expansion results in cell senescence inevitably, which will decrease the capability of MSCs, including differentiation ability, migration ability as well as regeneration ability (106, 107). Another issue that must be considered is the safety of MSCs transplantation. Although some studies had proved the efficacy of MSCs in DM, which had been listed in **Table 2**, the numbers of clinic studies and involved patients of MSCs therapy were limited, thus the efficacy was unsure and hardly applied in the clinic. Additionally, some clinic experiments of MSCs in other diseases demonstrated that MSCs will boost cancer growth. A study indicated the expression of VEGF in tumor cells as well as the activation of RhoA-GTPase and ERK1/2, were increased after human MSCs condition medium treatment (118). Another research reported that gastric cancer MSCs promoted immune escape by secreting IL-8, inducing programmed cell death ligand 1 (PD-L1) expression in gastric cancer cells (119). It seems that MSCs contribute to tumor cell growth and tumor development. The relationship between MSCs and tumor cells is still unknown, leaving a great challenge for MSCs therapy application.

**Table 2:** The clinic trials of MSCs therapy in DM.

MSCs origins	Number of patients	The key findings	Follow-up period(year)	years	references
BM-MSCs	30 (BM-MSCs: 10 BM-MNCs: 10 Control: 10)	Both BM-MSCs and BM-MNCs therapies in T2DM result in significant decreases in insulin dose requirement accompanied by improvement in insulin sensitivity and β-cells function	1	2017	(61)
Umbilical cord-MSCs	42 (UC-MSCs/BM-MNCs: 21Control: 21)	MSCs/MSCs treatment cause progressive reductions in insulin dose requirements and HbA1c levels and increased fasting C-peptide levels as well as AUC_C-Pep_	1	2016	(108)
WJ-MSCs	61 (WJ-MSCs:31 Control:30)	Blood glucose, glycosylated hemoglobin, C-peptide, homeostasis model assessment of pancreatic islet β−cell function, and incidence of diabetic complications in the MSCs group were significantly improved when compared with the control group during the 36 months follow−up in T2DM	3	2016	(109)
WJ-MSCs	12 (liraglutide+WJ-MSCs:6 liraglutide:6)	liraglutide treatment in combination with WJ-MSCs improves glucose metabolism and the β cell function in T2D patients	6 months	2016	(110)
Adipose-MSCs	20 (AD-MSCs:10 Control:10)	Variable and sustained improvement in mean fasting blood glucose(FBG), post-meal blood glucose(PBG), HbA1c, and serum C-peptide was noted after the treatment of insulin-secreting mesenchymal stromal cell.	2	2015	(111)
BM-MSCs	20 (MSCs:10 Insulin treatment:10)	Autologous MSC treatment of new-onset type 1 diabetes may be a safe and feasible strategy to intervene in the disease process and preserve β-cell function	1	2015	(112)
WJ-MSCs	6	Following transplantation, no immediate or delayed toxicity associated with the cell administration, and the levels of fasting C-peptide, the peak value and the area under the C-peptide release curve increased significantly within one month and remained high during the follow-up period	2	2015	(113)
Umbilical cord-MSCs	18	FBG and PBG were significantly reduced and plasma C-peptide levels and regulatory T (Treg) cell number were numerically higher after UMSC transfusion in T2D patients.	6 months	2014	(114)
WJ-MSCs	22	WJ-MSC transplantation decreased the level of HbA1c, increased the level of fasting C-peptide, decreased the FBG, 2h-postprandial blood glucose level, insulin requirement, and oral hypoglycemic drugs; and reduced the systemic inflammation and T lymphocyte counts in patients with T2DM	1	2014	(53)
WJ-MSCs	29 (WJ-MSCs:15 Control:14)	No reported acute or chronic side effects in the MSCs group compared with the control group, both the HbA1c and C peptide in MSCs group patients were significantly better than either pre-therapy values or control group patients during the follow-up period in T1DM.	2	2013	(115)
Placenta-MSCs	10	The mean levels of insulin and C-peptide at each time point in a total of 10 patients were higher and the renal function and cardiac function were improved after MSCs infusion, indicating that transplantation of placenta-MSC represents a simple, safe and effective therapeutic approach for T2D patients with islet cell dysfunction	1	2011	(116)
Adipose-MSCs	11	Transplantation of insulin-secreting cells that differentiated from AM-MSCs decreased insulin requirement and Hb1Ac levels and serum C-peptide levels were improved in T1D patients.	2	2010	(117)

### The Potential of MSCs Therapy in DN

**Figure 2:**
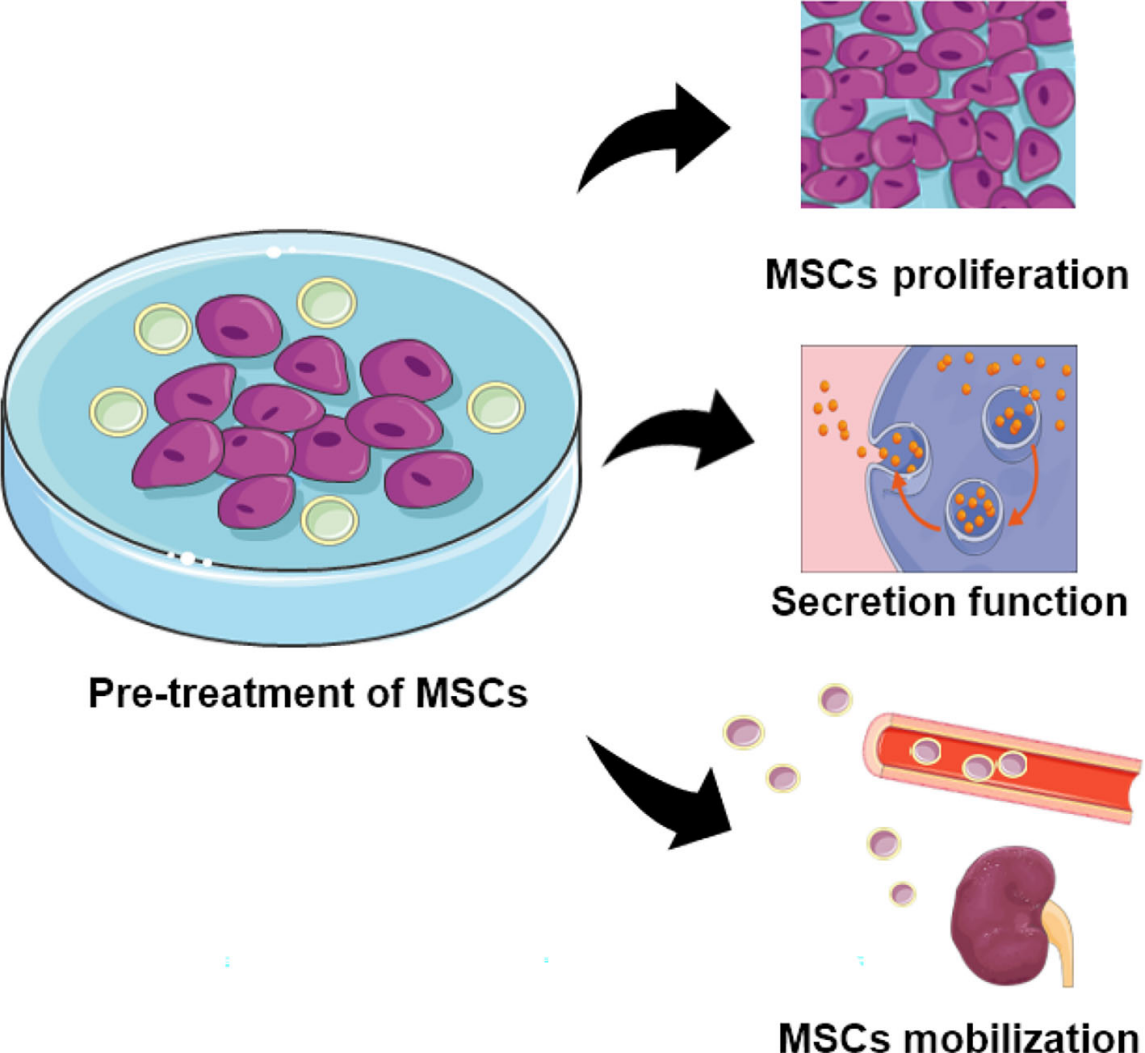
The function of MSCs pretreated with specific substances. Pre-treated MSCs demonstrate increased capability for proliferation, secretion, and localization.

MSCs from diverse donors with different capability as mentioned, the ability of MSCs from healthy people are superior to that from patients. While autologous MSCs with less immunological rejection shows better potential than MSCs from other individuals, which contradicted with the impaired regeneration and function of autologous MSCs (120, 121). Therefore, some research has focused on MSCs modification and co-culture to increase the MSC capacity in cell therapy. Pre-treatment of MSCs with specific substances as well as the growth environment had been revealed to enhance the MSCs therapeutic effect in DN. The general pathways had been shown in **Figure 2**.

The angiotensin-converting enzyme 2 (ACE2) plays a protective role in DN patients *via* degrading Ang II into Ang2-7, thus alleviating the detrimental effects of Ang II. Liu Q et al. found that ACE2-modified MSCs showed superior amelioration on glomerular fibrosis in DN compared to MSCs alone. After co-culturing ACE2 with MSCs, the expression of ACE2 was obviously higher and MSCs-ACE2 treatment groups showed reduced levels of collagen I as well as TGF-β mRNA and protein. The pre-treatment had diverse effects on the expression of angiotensin receptor (ATR). The injection of MSCs-ACE2 did no effect on the expression of AT1R, while the expression of AT2R increased; This increase in the MSCs-ACE2 group was greater than that in either the MSCs group or the ACE2 alone, which gives speculation that elevated AT2R is involved in the renal protective effect of MSCs-ACE2 treatment (122).

Melatonin (MT) is a neurohormone mainly secreted by the pineal and non-pineal cells and has demonstrated powerful antioxidative and anti‐inflammation properties for kidney diseases like acute kidney injury (AKI) as well as CDK. MSCs treated with MT also had a significant effect on DN treatment. Rashed et al. discovered that MSCs treated with MT showed positive effects in a DN model. Respectively, the increased TNF-α, and decreased TGF-ß, IL-10, and SOD corresponded with improved antioxidative, anti-fibrosis, and anti-inflammation effects. Additionally, MT pre-incubation significantly increased the cell proliferation of MSCs *in vitro* (123). Other research found that cellular prion protein (PrP^C^) mediated the functional recovery of MSCs. A team observed that MT-treated CKD-MSCs had a longer survival rate and alleviation of senescence. Furthermore, they found PrP^C^ was overexpressed after MT treatment. Enhanced mitochondrial activity, as well as MSCs functional recovery, corresponded with MT treatment. PrP^C^ knockdown significantly neutralized the benefits from MT-MSCs treatment, suggesting the alleviation effects were mediated by PrP^C^ (124). Focusing on the MSCs functional rescue research, it was shown that MSCs treated with MT-derived exosomes had been discovered to transfer microRNAs to stimulate the increase of PrP^C^, thereby recovering MSCs functions (125). The team developed and finished a complete logical story of how MT affects the function of MSCs. MT possesses the ability to enhance MSCs capabilities and demonstrates the potential for constructive effects with MSCs-based therapy in DN. Additionally, factors including clinical drugs and other biological hormones were involved in PrP^C^ expression (126–128), providing a promising approach for PrP^C^ expression to enhance MSCs function.

Umbilical cord extract, namely Wharton’s jelly extract supernatant (WJs), which contains several types of biologically active substances including growth factors, cytokines, extracellular matrixes, and exosomes, provides a suitable survival environment to maintain MSCs properties. By culturing with WJ, the morphology, proliferative ability, and cell mobilization of BM-MSCs in a DN model increased to a large extent. Meanwhile, the mitochondrial degeneration and abnormal expansion of the endoplasmic reticulum (ER) were improved as well. As for the mechanism, exosomes secreted by WJ might be the key factor to activate DM-MSCs, since WJ-derived exosomes showed similar effects on MSC function compared with WJ (129).

## Conclusion

Increased prevalence and low therapeutic effects of diabetes make kidney impairment inevitable. Similarly, ineffective treatment of DN often ends with CKD and ESRD, which lead to kidney transplantation and even death. The MSCs-based cell therapy brings a prospective treatment for DM as well as DN. It had been reported that MSCs were involved in blood glucose reduction, anti-inflammation, anti-fibrosis, podocyte protection, and pro-angiogenesis processes in DN. Furthermore, researchers investigated the mechanisms of MSC therapy and found that exosomes play a significant role in MSC therapeutic effects. Exosomes serve as a vehicle, transmitting a variety of substances from MSCs to recipient cells, especially microRNAs; This may confer positive effects to recipient cells. Hopefully, autologous MSCs with little immunological rejection is of more significance than MSCs from other origins in DN treatment. However, kidney injury is regularly accompanied by impairment of MSCs function, resulting in lower therapeutic effectiveness of autologous MSCs. Studies concerning MSCs functional recovery emerged under this situation. Factors including clinical drugs and hormones have been involved in the MSCs functional recovery *via* improving MSCs growth and secretory capabilities.

Even with some challenges for MSCs therapy in clinic application, MSCs-based cell therapy offers a bright future for DN treatment. Exosomes from MSCs as well as pre-treatment of MSCs can be regarded as a key breakthrough for improving therapeutic efficiency. More clinical trials are required to identify the efficacy of MSCs in DN.

## Author Contributions

L-QY: manuscript writing and approving final version of manuscript. YW: study conduct, data analysis, and manuscript writing. S-KS, BG, FL, M-HZ, L-ML, Q-SX and UM: data analysis. All authors contributed to the article and approved the submitted version.

## Funding

This work was supported by funding from the National Natural Science Foundation of China (Nos. 81770881 and 82070910). Key R & D plan of Hunan Province(2020SK2078).

## Conflict of Interest

The authors declare that the research was conducted in the absence of any commercial or financial relationships that could be construed as a potential conflict of interest.
